# 10α-Hy­droxy-4,9-dimethyl-13-[(pyrrol­idin-1-yl)meth­yl]-3,8,15-trioxatetra­cyclo­[10.3.0.0^2,4^.0^7,9^]penta­decan-14-one

**DOI:** 10.1107/S1600536811024688

**Published:** 2011-06-30

**Authors:** Mohamed Moumou, Ahmed Benharref, Moha Berraho, Daniel Avignant, Abdelghani Oudahmane, Mohamed Akssira

**Affiliations:** a Laboratoire de Chimie Biomoléculaire, Substances Naturelles et Réactivité, URAC 16, Faculté des Sciences Semlalia, BP 2390, Bd My Abdellah,40000 Marrakech, Morocco; b Laboratoire des Matériaux Inorganiques, Université Blaise Pascal, UMR CNRS 6002, 24 Avenue des Landais, 63177 Aubière, France; c Laboratoire de Chimie Bioorganique et Analytique, URAC 22, BP 146, FSTM, Université Hassan II, Mohammedia-Casablanca 20810 Mohammedia, Morocco

## Abstract

The title compound, C_19_H_29_NO_5_, was synthesized from 9α-hy­droxy­parthenolide (9α-hy­droxy-4,8-dimethyl-12-methyl­ene-3,14-dioxatricyclo­[9.3.0.0^2,4^]tetra­dec-7-en-13-one), which was isolated from the chloro­form extract of the aerial parts of *Anvillea radiata*. The mol­ecule is built up from two fused five- and ten-membered rings with the (pyrrolidin-4-yl)methyl group as a substituent. The two five-membered ring display the same envelope conformations, whereas the ten-membered ring adopts an approximate chair–chair conformation. The dihedral angle between the ten-membered ring and the lactone ring is 21.81 (9)°. An intra­molecular O—H⋯N hydrogen bond stabilizes the mol­ecular conformation. In the crystal, inter­molecular C—H⋯O inter­actions link the mol­ecules into chains parallel to the *c* axis.

## Related literature

For background to the medicinal uses of the plant *Anvillea radiata*, see: El Hassany *et al.* (2004 ▶). For reactivity of this sesquiterpene see: Der-Ren *et al.* (2006 ▶); Neelakantan *et al.* (2009 ▶); Neukirch *et al.* (2003 ▶). For ring puckering parameters, see: Cremer & Pople (1975 ▶). For conformations of ten-membered rings, see: Castaneda-Acosta *et al.* (1997 ▶). For related structures, see: Moumou *et al.* (2010 ▶); Watson & Zabel (1982 ▶).
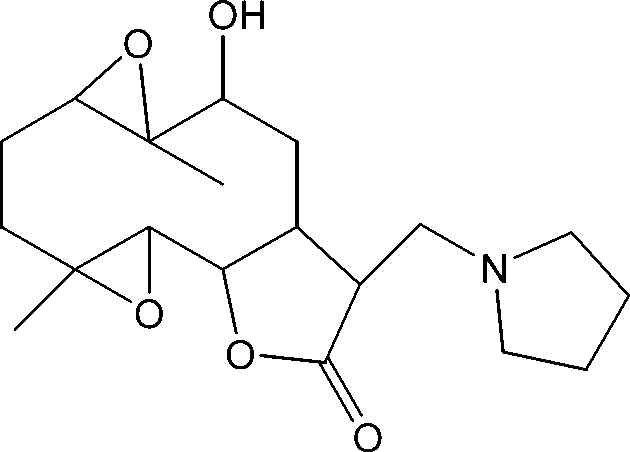


## Experimental

### 

#### Crystal data

C_19_H_29_NO_5_
*M* *_r_* = 351.43
Orthorhombic, 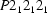

*a* = 8.0714 (2) Å
*b* = 10.4571 (3) Å
*c* = 21.5816 (8) Å
*V* = 1821.56 (10) Å^3^
*Z* = 4
Mo *K*α radiation
μ = 0.09 mm^−1^
*T* = 298 K
0.89 × 0.46 × 0.21 mm

#### Data collection

Bruker APEXII CCD area-detector diffractometer
8707 measured reflections
2122 independent reflections
1660 reflections with *I* > 2σ(*I*)
*R*_int_ = 0.034

#### Refinement

*R*[*F* ^2^ > 2σ(*F* ^2^)] = 0.038
*wR*(*F* ^2^) = 0.098
*S* = 1.03
2122 reflections
229 parameters
H-atom parameters constrained
Δρ_max_ = 0.17 e Å^−3^
Δρ_min_ = −0.15 e Å^−3^

### 

Data collection: *APEX2* (Bruker, 2005 ▶); cell refinement: *APEX2* and *SAINT* (Bruker, 2005 ▶); data reduction: *SAINT*; program(s) used to solve structure: *SHELXS97* (Sheldrick, 2008 ▶); program(s) used to refine structure: *SHELXL97* (Sheldrick, 2008 ▶); molecular graphics: *ORTEP-3 for Windows* (Farrugia, 1997 ▶) and *PLATON* (Spek, 2009 ▶); software used to prepare material for publication: *WinGX* (Farrugia, 1999 ▶).

## Supplementary Material

Crystal structure: contains datablock(s) I, global. DOI: 10.1107/S1600536811024688/om2443sup1.cif

Structure factors: contains datablock(s) I. DOI: 10.1107/S1600536811024688/om2443Isup2.hkl

Supplementary material file. DOI: 10.1107/S1600536811024688/om2443Isup3.cml

Additional supplementary materials:  crystallographic information; 3D view; checkCIF report

## Figures and Tables

**Table 1:** Hydrogen-bond geometry (Å, °)

*D*—H⋯*A*	*D*—H	H⋯*A*	*D*⋯*A*	*D*—H⋯*A*
O3—H3⋯N	0.82	2.04	2.851 (2)	172
C9—H9⋯O4^i^	0.98	2.38	3.260 (2)	149
C10—H10⋯O2^ii^	0.98	2.46	3.392 (3)	158
C19—H19*B*⋯O5^iii^	0.97	2.59	3.357 (3)	136

Symmetry codes: (i) 
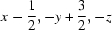
; (ii) 
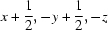
; (iii) 
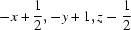
.

**Fig. 1.**
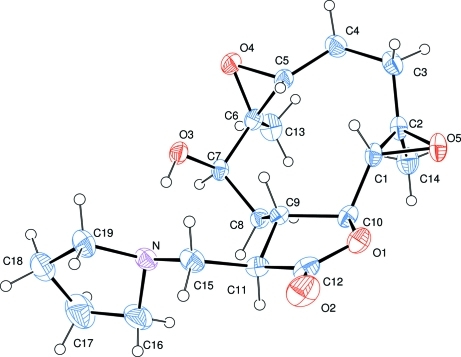
Molecular structure of the title compound with the atom-labelling scheme. Displacement ellipsoids are drawn at the 30% probability level. H atoms are represented as small spheres of arbitrary radii.

**Fig. 2.**
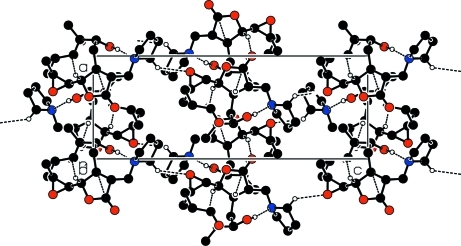
Packing view showing the C–H···O and O–H···N hydrogen bonds as dashed lines. H atoms not involved in hydrogen bonding have been omitted for clarity.

**Fig. 3.**
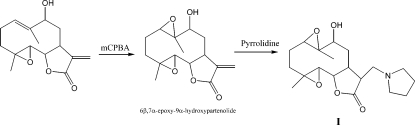
Synthesis of the title compound.

## supplementary crystallographic information

### Comment

Our work lies within the framework of the valorization of medicinals plants and concerning the *Anvillea radiata*. The main constituent of the chloroform extract of aerial parts of this plant is 9α-hydroxypartenolide (El Hassany *et al.*, 2004). The reactivity of this sesquiterpene lactone and its derivatives has been the subject of several studies (Neukirch *et al.*, 2003; Der-Ren *et al.*, 2006; Neelakantan *et al.*, 2009), in order to prepare products with a high added value that can be used in the pharmacology industry. In this context, we have treated the 9α-hydroxypartenolide with one equivalent of *meta*-chloroperbenzoic acid (mCPBA), as we have done for its isomer the 9β-hydroxypartenolide (Moumou *et al.*,2010), and we got the 6β,7α-epoxy-9α-hydroxypartenolide in a yield of 75% (see Figure 3). This latter was treated with an equivalent of pyrrolodine and gives the title compound (I) in a yield of 95%. The molecule contains two fused rings which exhibit different conformations with a pyrolidin ring as a substituent to the lactone ring. The molecular structure of (I), Fig. 1, shows that the two five membered rings adopt an envelope conformation, as indicated by Cremer & Pople (1975) puckering parameters Q = 0.298 (2) Å and φ = 77.5 (4)° for the lactone ring and Q = 0.362 (3) Å, φ2 = 6.4 (6)° for the pyrolidin ring. The ten-membered ring displays an approximate chair-chair conformation, this is the typical conformation observed for other sesquiterpenes lactones (Moumou *et al.*, 2010; Watson & Zabel, 1982; Castaneda-Acosta *et al.*, 1997). In the crystal structure, molecules are linked into supramolecular chains (Fig. 2) parallel to the *c* axis by C—H···O hydrogen bonds (Table 1). In addition an intramolecular O—H···N hydrogen bond is also observed.

### Experimental

The mixture of 6β,7α-epoxy-9α hydoxy partenolide (0.5 g, 2 mmol) and one equivalent of pyrolidine in EtOH (20 ml) was stirred for one night at room temperature. The next day the reaction was stopped by adding water (10 ml) and extracted three times with ethyl acetate (3 *x* 20 ml). The combined organic layers were dried over anhydrous MgSO_4_, filtered and concentrated under vacuum to give 666 mg (1.9 mmol) of solid which was recrystallized in ethyl acetate.

### Refinement

All H atoms were fixed geometrically and treated as riding with C—H = 0.96 Å (methyl), 0.97 Å (methylene), 0. 98Å (methine) with *U*_iso_(H) = 1.2*U*_eq_ (methylene, methine) or *U*_iso_(H) = 1.5*U*_eq_ (methyl, OH). In the absence of significant anomalous scattering, the absolute configuration could not be reliably determined and thus 1295 Friedel pairs were merged and any references to the Flack parameter were removed.

### Crystal data

**Table:**

C_19_H_29_NO_5_	*F*(000) = 760
*M**_r_* = 351.43	*D*_x_ = 1.281 Mg m^−^^3^
Orthorhombic, *P*2_1_2_1_2_1_	Mo *K*α radiation, λ = 0.71073 Å
Hall symbol: P 2ac 2ab	Cell parameters from 3417 reflections
*a* = 8.0714 (2) Å	θ = 2.7–26.4°
*b* = 10.4571 (3) Å	µ = 0.09 mm^−^^1^
*c* = 21.5816 (8) Å	*T* = 298 K
*V* = 1821.56 (10) Å^3^	Prism, colourless
*Z* = 4	0.89 × 0.46 × 0.21 mm

### Data collection

**Table:**

Bruker APEXII CCD area-detector diffractometer	1660 reflections with *I* > 2σ(*I*)
Radiation source: fine-focus sealed tube	*R*_int_ = 0.034
graphite	θ_max_ = 26.4°, θ_min_ = 2.7°
φ and ω scans	*h* = −7→10
8707 measured reflections	*k* = −13→10
2122 independent reflections	*l* = −26→25

### Refinement

**Table:**

Refinement on *F*^2^	Primary atom site location: structure-invariant direct methods
Least-squares matrix: full	Secondary atom site location: difference Fourier map
*R*[*F*^2^ > 2σ(*F*^2^)] = 0.038	Hydrogen site location: inferred from neighbouring sites
*wR*(*F*^2^) = 0.098	H-atom parameters constrained
*S* = 1.03	*w* = 1/[σ^2^(*F*_o_^2^) + (0.0589*P*)^2^] where *P* = (*F*_o_^2^ + 2*F*_c_^2^)/3
2122 reflections	(Δ/σ)_max_ < 0.001
229 parameters	Δρ_max_ = 0.17 e Å^−^^3^
0 restraints	Δρ_min_ = −0.15 e Å^−^^3^

### Special details

**Table:**

Geometry. All s.u.'s (except the s.u. in the dihedral angle between two l.s. planes) are estimated using the full covariance matrix. The cell s.u.'s are taken into account individually in the estimation of s.u.'s in distances, angles and torsion angles; correlations between s.u.'s in cell parameters are only used when they are defined by crystal symmetry. An approximate (isotropic) treatment of cell s.u.'s is used for estimating s.u.'s involving l.s. planes.

### Fractional atomic coordinates and isotropic or equivalent isotropic displacement parameters (Å^2^)

**Table:**

	*x*	*y*	*z*	*U*_iso_*/*U*_eq_	
C1	0.2294 (3)	0.5169 (2)	0.08783 (11)	0.0361 (6)	
H1	0.1851	0.5945	0.0685	0.043*	
C2	0.3205 (3)	0.5410 (2)	0.14572 (11)	0.0399 (6)	
C3	0.3293 (3)	0.6785 (2)	0.16551 (12)	0.0483 (7)	
H3A	0.3354	0.6820	0.2104	0.058*	
H3B	0.2280	0.7212	0.1530	0.058*	
C4	0.4776 (3)	0.7513 (3)	0.13836 (13)	0.0491 (7)	
H4A	0.4573	0.8423	0.1424	0.059*	
H4B	0.5754	0.7311	0.1626	0.059*	
C5	0.5120 (3)	0.7213 (2)	0.07148 (12)	0.0377 (6)	
H5	0.4129	0.7113	0.0457	0.045*	
C6	0.6598 (3)	0.6526 (2)	0.04855 (11)	0.0361 (5)	
C7	0.6555 (3)	0.5740 (2)	−0.01081 (10)	0.0336 (5)	
H7	0.7700	0.5517	−0.0214	0.040*	
C8	0.5592 (3)	0.4481 (2)	−0.00134 (11)	0.0351 (5)	
H8A	0.6027	0.3845	−0.0297	0.042*	
H8B	0.5792	0.4177	0.0405	0.042*	
C9	0.3708 (3)	0.4590 (2)	−0.01138 (10)	0.0325 (5)	
H9	0.3454	0.5497	−0.0179	0.039*	
C10	0.2637 (3)	0.4126 (2)	0.04320 (12)	0.0367 (6)	
H10	0.3146	0.3389	0.0639	0.044*	
C11	0.2981 (3)	0.3852 (2)	−0.06591 (12)	0.0410 (6)	
H11	0.3523	0.3016	−0.0685	0.049*	
C12	0.1199 (3)	0.3663 (2)	−0.04627 (14)	0.0491 (7)	
C13	0.7995 (3)	0.6124 (3)	0.09028 (13)	0.0549 (8)	
H13A	0.9032	0.6363	0.0718	0.082*	
H13B	0.7963	0.5214	0.0959	0.082*	
H13C	0.7881	0.6539	0.1297	0.082*	
C14	0.4501 (3)	0.4522 (3)	0.17126 (13)	0.0557 (7)	
H14A	0.4431	0.4509	0.2157	0.084*	
H14B	0.5580	0.4814	0.1590	0.084*	
H14C	0.4321	0.3675	0.1554	0.084*	
C15	0.3068 (3)	0.4494 (3)	−0.12859 (12)	0.0469 (6)	
H15A	0.2480	0.3971	−0.1585	0.056*	
H15B	0.2506	0.5312	−0.1262	0.056*	
C16	0.5619 (4)	0.3520 (3)	−0.16867 (15)	0.0686 (9)	
H16A	0.4884	0.2950	−0.1910	0.082*	
H16B	0.6043	0.3079	−0.1324	0.082*	
C17	0.7000 (5)	0.3955 (4)	−0.2091 (2)	0.0935 (13)	
H17A	0.7218	0.3332	−0.2414	0.112*	
H17B	0.8003	0.4078	−0.1851	0.112*	
C18	0.6423 (4)	0.5219 (3)	−0.23718 (14)	0.0664 (9)	
H18A	0.7210	0.5895	−0.2283	0.080*	
H18B	0.6301	0.5143	−0.2817	0.080*	
C19	0.4779 (4)	0.5494 (3)	−0.20714 (12)	0.0553 (7)	
H19A	0.4686	0.6394	−0.1968	0.066*	
H19B	0.3875	0.5265	−0.2346	0.066*	
N	0.4750 (3)	0.47021 (19)	−0.15073 (9)	0.0435 (5)	
O1	0.1047 (2)	0.37802 (16)	0.01525 (9)	0.0474 (5)	
O2	0.0011 (3)	0.3449 (2)	−0.07852 (11)	0.0696 (6)	
O3	0.5907 (2)	0.64734 (16)	−0.06008 (8)	0.0415 (4)	
H3	0.5656	0.5999	−0.0888	0.062*	
O4	0.6462 (2)	0.78993 (16)	0.04219 (8)	0.0460 (5)	
O5	0.1545 (2)	0.48703 (17)	0.14653 (8)	0.0503 (5)	

### Atomic displacement parameters (Å^2^)

**Table:**

	*U*^11^	*U*^22^	*U*^33^	*U*^12^	*U*^13^	*U*^23^
C1	0.0278 (11)	0.0437 (13)	0.0369 (13)	−0.0005 (11)	0.0061 (10)	0.0094 (10)
C2	0.0353 (13)	0.0507 (14)	0.0338 (13)	−0.0003 (12)	0.0068 (10)	0.0073 (12)
C3	0.0521 (15)	0.0586 (16)	0.0340 (14)	0.0070 (13)	0.0051 (12)	−0.0027 (12)
C4	0.0538 (16)	0.0513 (15)	0.0423 (16)	−0.0036 (13)	0.0025 (13)	−0.0083 (12)
C5	0.0378 (13)	0.0365 (12)	0.0389 (14)	−0.0073 (12)	0.0015 (11)	0.0009 (11)
C6	0.0270 (11)	0.0401 (13)	0.0411 (14)	−0.0065 (11)	−0.0007 (10)	0.0050 (11)
C7	0.0269 (11)	0.0398 (12)	0.0341 (13)	0.0029 (11)	0.0037 (10)	0.0065 (10)
C8	0.0352 (12)	0.0341 (11)	0.0359 (13)	0.0025 (11)	0.0024 (10)	0.0030 (10)
C9	0.0337 (11)	0.0284 (10)	0.0354 (12)	0.0000 (10)	−0.0004 (10)	0.0027 (10)
C10	0.0304 (12)	0.0348 (12)	0.0450 (15)	−0.0028 (10)	−0.0001 (11)	0.0096 (11)
C11	0.0444 (14)	0.0343 (12)	0.0442 (15)	0.0005 (12)	−0.0029 (12)	−0.0032 (11)
C12	0.0517 (17)	0.0366 (13)	0.0591 (19)	−0.0096 (14)	−0.0047 (15)	0.0015 (13)
C13	0.0385 (15)	0.077 (2)	0.0487 (17)	−0.0019 (14)	−0.0071 (13)	0.0033 (14)
C14	0.0566 (16)	0.0671 (17)	0.0434 (16)	0.0044 (16)	−0.0038 (13)	0.0116 (15)
C15	0.0485 (15)	0.0516 (14)	0.0405 (15)	0.0041 (14)	−0.0047 (12)	−0.0067 (12)
C16	0.079 (2)	0.0623 (18)	0.064 (2)	0.0231 (18)	0.0107 (19)	−0.0128 (16)
C17	0.083 (3)	0.116 (3)	0.082 (3)	0.026 (2)	0.027 (2)	−0.008 (2)
C18	0.070 (2)	0.081 (2)	0.0476 (18)	−0.0066 (19)	0.0082 (16)	−0.0081 (16)
C19	0.0636 (17)	0.0695 (18)	0.0327 (14)	0.0029 (17)	−0.0035 (13)	−0.0014 (14)
N	0.0512 (13)	0.0459 (12)	0.0333 (11)	0.0082 (11)	−0.0027 (10)	−0.0041 (9)
O1	0.0366 (10)	0.0480 (10)	0.0576 (12)	−0.0131 (8)	0.0013 (9)	0.0040 (9)
O2	0.0568 (13)	0.0737 (14)	0.0784 (16)	−0.0238 (12)	−0.0205 (12)	−0.0018 (12)
O3	0.0507 (10)	0.0409 (8)	0.0327 (10)	−0.0062 (9)	−0.0004 (8)	0.0074 (7)
O4	0.0488 (10)	0.0406 (9)	0.0485 (11)	−0.0146 (8)	0.0053 (9)	0.0000 (8)
O5	0.0399 (9)	0.0666 (12)	0.0445 (11)	−0.0044 (9)	0.0147 (8)	0.0106 (9)

### Geometric parameters (Å, °)

**Table:**

C1—O5	1.438 (3)	C10—H10	0.9800
C1—C2	1.472 (3)	C11—C15	1.512 (4)
C1—C10	1.481 (3)	C11—C12	1.512 (4)
C1—H1	0.9800	C11—H11	0.9800
C2—O5	1.454 (3)	C12—O2	1.206 (3)
C2—C3	1.501 (4)	C12—O1	1.339 (3)
C2—C14	1.504 (4)	C13—H13A	0.9600
C3—C4	1.535 (4)	C13—H13B	0.9600
C3—H3A	0.9700	C13—H13C	0.9600
C3—H3B	0.9700	C14—H14A	0.9600
C4—C5	1.503 (4)	C14—H14B	0.9600
C4—H4A	0.9700	C14—H14C	0.9600
C4—H4B	0.9700	C15—N	1.455 (3)
C5—O4	1.445 (3)	C15—H15A	0.9700
C5—C6	1.478 (3)	C15—H15B	0.9700
C5—H5	0.9800	C16—N	1.473 (3)
C6—O4	1.446 (3)	C16—C17	1.487 (5)
C6—C13	1.503 (3)	C16—H16A	0.9700
C6—C7	1.523 (3)	C16—H16B	0.9700
C7—O3	1.412 (3)	C17—C18	1.526 (5)
C7—C8	1.543 (3)	C17—H17A	0.9700
C7—H7	0.9800	C17—H17B	0.9700
C8—C9	1.540 (3)	C18—C19	1.504 (4)
C8—H8A	0.9700	C18—H18A	0.9700
C8—H8B	0.9700	C18—H18B	0.9700
C9—C11	1.525 (3)	C19—N	1.473 (3)
C9—C10	1.540 (3)	C19—H19A	0.9700
C9—H9	0.9800	C19—H19B	0.9700
C10—O1	1.464 (3)	O3—H3	0.8200
			
O5—C1—C2	59.96 (15)	C9—C10—H10	111.2
O5—C1—C10	119.5 (2)	C15—C11—C12	110.7 (2)
C2—C1—C10	125.8 (2)	C15—C11—C9	116.62 (19)
O5—C1—H1	113.7	C12—C11—C9	102.5 (2)
C2—C1—H1	113.7	C15—C11—H11	108.9
C10—C1—H1	113.7	C12—C11—H11	108.9
O5—C2—C1	58.87 (15)	C9—C11—H11	108.9
O5—C2—C3	114.3 (2)	O2—C12—O1	121.1 (3)
C1—C2—C3	115.4 (2)	O2—C12—C11	128.2 (3)
O5—C2—C14	113.4 (2)	O1—C12—C11	110.7 (2)
C1—C2—C14	123.6 (2)	C6—C13—H13A	109.5
C3—C2—C14	117.0 (2)	C6—C13—H13B	109.5
C2—C3—C4	113.8 (2)	H13A—C13—H13B	109.5
C2—C3—H3A	108.8	C6—C13—H13C	109.5
C4—C3—H3A	108.8	H13A—C13—H13C	109.5
C2—C3—H3B	108.8	H13B—C13—H13C	109.5
C4—C3—H3B	108.8	C2—C14—H14A	109.5
H3A—C3—H3B	107.7	C2—C14—H14B	109.5
C5—C4—C3	114.0 (2)	H14A—C14—H14B	109.5
C5—C4—H4A	108.7	C2—C14—H14C	109.5
C3—C4—H4A	108.7	H14A—C14—H14C	109.5
C5—C4—H4B	108.7	H14B—C14—H14C	109.5
C3—C4—H4B	108.7	N—C15—C11	113.8 (2)
H4A—C4—H4B	107.6	N—C15—H15A	108.8
O4—C5—C6	59.31 (14)	C11—C15—H15A	108.8
O4—C5—C4	117.1 (2)	N—C15—H15B	108.8
C6—C5—C4	124.9 (2)	C11—C15—H15B	108.8
O4—C5—H5	114.6	H15A—C15—H15B	107.7
C6—C5—H5	114.6	N—C16—C17	104.8 (3)
C4—C5—H5	114.6	N—C16—H16A	110.8
O4—C6—C5	59.20 (15)	C17—C16—H16A	110.8
O4—C6—C13	113.1 (2)	N—C16—H16B	110.8
C5—C6—C13	122.7 (2)	C17—C16—H16B	110.8
O4—C6—C7	117.0 (2)	H16A—C16—H16B	108.9
C5—C6—C7	121.7 (2)	C16—C17—C18	105.6 (3)
C13—C6—C7	111.7 (2)	C16—C17—H17A	110.6
O3—C7—C6	110.42 (18)	C18—C17—H17A	110.6
O3—C7—C8	112.11 (19)	C16—C17—H17B	110.6
C6—C7—C8	111.14 (18)	C18—C17—H17B	110.6
O3—C7—H7	107.7	H17A—C17—H17B	108.7
C6—C7—H7	107.7	C19—C18—C17	105.3 (3)
C8—C7—H7	107.7	C19—C18—H18A	110.7
C9—C8—C7	114.57 (19)	C17—C18—H18A	110.7
C9—C8—H8A	108.6	C19—C18—H18B	110.7
C7—C8—H8A	108.6	C17—C18—H18B	110.7
C9—C8—H8B	108.6	H18A—C18—H18B	108.8
C7—C8—H8B	108.6	N—C19—C18	105.2 (2)
H8A—C8—H8B	107.6	N—C19—H19A	110.7
C11—C9—C10	102.40 (17)	C18—C19—H19A	110.7
C11—C9—C8	116.8 (2)	N—C19—H19B	110.7
C10—C9—C8	115.03 (19)	C18—C19—H19B	110.7
C11—C9—H9	107.3	H19A—C19—H19B	108.8
C10—C9—H9	107.3	C15—N—C19	111.7 (2)
C8—C9—H9	107.3	C15—N—C16	113.9 (2)
O1—C10—C1	106.59 (19)	C19—N—C16	104.3 (2)
O1—C10—C9	104.76 (18)	C12—O1—C10	110.54 (19)
C1—C10—C9	111.73 (18)	C7—O3—H3	109.5
O1—C10—H10	111.2	C5—O4—C6	61.49 (14)
C1—C10—H10	111.2	C1—O5—C2	61.17 (15)

### Hydrogen-bond geometry (Å, °)

**Table:**

*D*—H···*A*	*D*—H	H···*A*	*D*···*A*	*D*—H···*A*
O3—H3···N	0.82	2.04	2.851 (2)	172
C9—H9···O4^i^	0.98	2.38	3.260 (2)	149
C10—H10···O2^ii^	0.98	2.46	3.392 (3)	158
C19—H19B···O5^iii^	0.97	2.59	3.357 (3)	136

Symmetry codes:  (i) *x*−1/2, −*y*+3/2, −*z*; (ii) *x*+1/2, −*y*+1/2, −*z*; (iii) −*x*+1/2, −*y*+1, *z*−1/2.
